# Supplementary material for: Accurate quantum-centric simulations of supramolecular interactions
Source: Res Sq. 2025 Mar 19:rs.3.rs-5566874. Preprint. [Version 1] doi: 10.21203/rs.3.rs-5566874/v1 (PMC11957199; doi:10.21203/rs.3.rs-5566874/v1)
Supplement: Supplement 1 [file NIHPPrs5566874v1-supplement-1.pdf]

# SUPPLEMENTARY INFORMATION: ACCURATE QUANTUM-CENTRIC SIMULATIONS OF SUPRAMOLECULAR INTERACTIONS

## I. GEOMETRIES OF POTENTIAL ENERGY SURFACES

The PES for the water dimer is calculated for distances between two oxygen atoms ranging between 1.400 and 3.500 Å. We distribute the points for water dimer PES as 1.400, 1.500, 1.600, 1.700, 1.800, 1.900, 1.962, 2.000, 2.100, 2.200, 2.300, 2.400, 2.500, 3.000, and 3.500 Å. All of water dimer simulations are done for all of these distances. The PES for the methane dimer is calculated for the distances between two carbon atoms ranging between 2.500 and 6.000 Å. We distribute the points for methane dimer PES as 2.500, 2.750, 3.000, 3.167, 3.334, 3.500, 3.667, 3.834, 4.000, 4.250, 4.500, 4.750, 5.000, and 6.000 Å. To calculate the total energy of unbound dimer we utilize the distance of 48.000 Å for both water and methane dimers. All of CASCI (16e,16o), CCSD, CCSD(T), CCSD (16e,16o), and CCSD(T) (16e,16o) simulations of methane dimer as well as SQD (16e,16o) simulations with $|\tilde{\chi}_b| = 20.0 \cdot 10^3$ are done for all of the distances described earlier. The methane dimer CASCI (16e,16o) simulations and SQD (16e,16o) simulations with $|\tilde{\chi}_b| = 20.0 \cdot 10^3$ are also performed for an additional distance of 3.638 Å. The SQD (16e,16o) energy extrapolations using $|\tilde{\chi}_b|$ of $9.0 \cdot 10^3$, $11.0 \cdot 10^3$, and $14.0 \cdot 10^3$ are done for 4.000, 4.250, 4.500, 4.750, 5.000, 6.000, and 48.000 Å distances. In the case of HCI (16e,24o) simulations of the methane dimer, we use only distances of 3.638, 3.667, 3.750, 3.834, and 3.900 Å. In case of SQD (16e,24o) simulations we only use the distance of 3.638 Å. All calculations are done as single-point energy calculations with no geometry optimizations. To produce the geometries studies in this work, we start from the equilibrium geometries and change the distance between the centers of the monomers, with the geometries of the individual monomers fixed.

## II. DETAILS OF HCI CALCULATIONS

In HCI simulations instead of generating all of the single and double excitations one generates only those single and double excitations that correspond to Hamiltonian matrix elements exceeding a threshold $\varepsilon$. In HCI $\varepsilon_1$ controls which determinants will be included in the variational wave function. In our HCI calculations, we use values of $\varepsilon_1$ equal to $5 \cdot 10^{-6}$ (during initial variational steps) and $1 \cdot 10^{-6}$ (during later variational steps). We do not

use the non-variational perturbative correction in our HCI calculations. Hence, our HCI calculations are fully variational, which allows for more appropriate comparison between the SQD and HCI results.

## III. EFFECT OF NUMBER OF SAMPLES ON TOTAL ENERGY IN SQD(16E,16O)

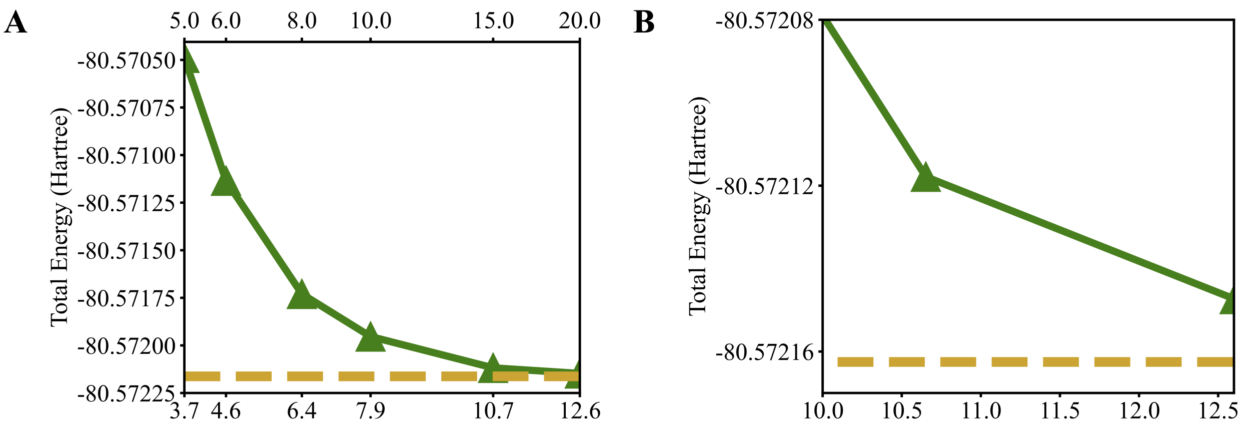


Figure S1. Total energy of methane dimer predicted with SQD (16e,16o) at 3.638 Å distance between the monomers as the function of $d \cdot 10^7$. (A) the entire range of $d$, and (B) a magnified region with largest values of $d$, highlighted in panel (A) as a black box. The secondary x-axis demonstrates the value of $|\widetilde{\chi}_b| \cdot 10^3$ producing the given value of $d \cdot 10^7$. Solid green line with triangular markers shows SQD (16e,16o) results. Horizontal dashed light brown line indicates the total energy from CASCI (16e,16o) calculation.

## IV. PES OF METHANE DIMER INCLUDING THE REPULSIVE REGION

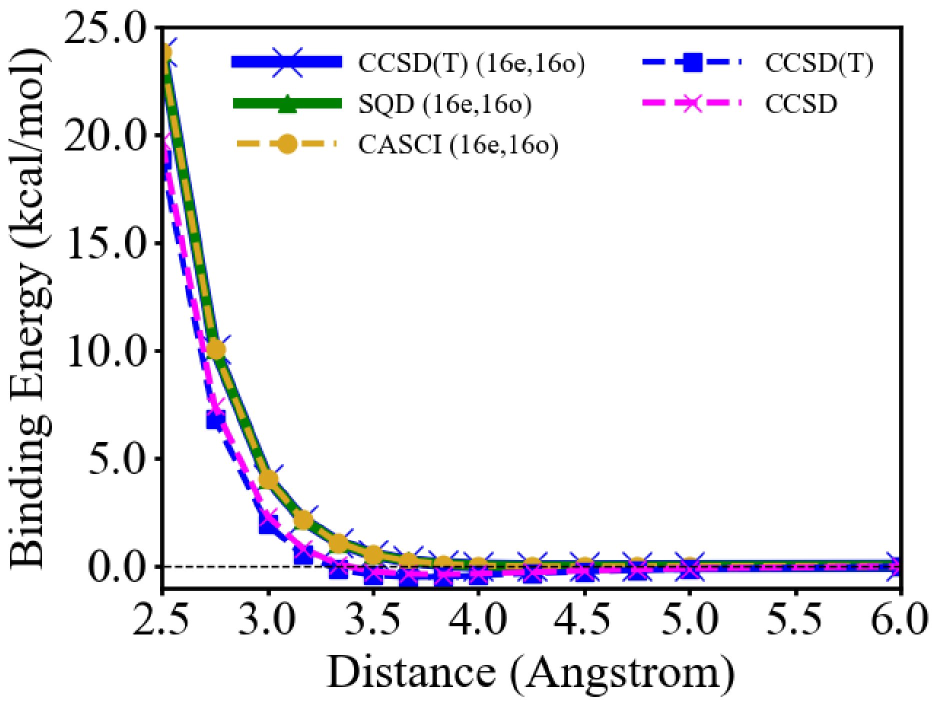


Figure S2. Binding energies of the methane dimer along its PES, where the distances between the centers of methane molecules range between 2.500 and 6.000 Å. Active space simulations are performed with (16e,16o). Light brown, orange, and blue dashed lines with circle markers depict PES calculated with CASCI, CCSD, and CCSD(T) methods, respectively. Solid yellow line with triangular markers depicts the PES calculated with the SQD method. Solid blue line represents CCSD(T) calculations using an active space. Black horizontal dashed line indicates the zero value of the binding energy.
